# Combined Lipidomics and Network Pharmacology Study of Protective Effects of *Salvia miltiorrhiza* against Blood Stasis Syndrome

**DOI:** 10.1155/2021/5526778

**Published:** 2021-03-19

**Authors:** Yidian Jin, Zhiru Xie, Shasha Li, Xiangyu Zeng, Leqi Wang, Ping Hu, Hongyang Zhang, Xue Xiao

**Affiliations:** ^1^School of Chemistry and Molecular Engineering, East China University of Science and Technology, Shanghai 200237, China; ^2^The Second Affiliated Hospital of Guangzhou University of Chinese Medicine, Guangzhou 510120, China; ^3^Institute of Traditional Chinese Medicine, Guangdong Pharmaceutical University, Guangzhou 510006, China; ^4^Shanghai Key Laboratory of New Drug Design, School of Pharmacy, East China University of Science and Technology, Shanghai 200237, China

## Abstract

Blood stasis syndrome (BSS) is one of the most common symptoms of cardiovascular diseases (CVDs) in traditional Chinese medicine (TCM) theory. Previous studies have identified that *Salvia miltiorrhiza* (Danshen) has beneficial effects on BSS, but there is no relevant research from the perspective of lipidomics to study the mechanism of Danshen against BSS since hyperlipidemia has been the widely accepted risk factor of CVDs. In this study, lipidomics technology combined with network pharmacology was applied to investigate the pathological mechanism of BSS and the protective effects of Danshen. The lipidomics profiling based on the UPLC-QTOF-MS analysis method was applied to identify the differential metabolites in the plasma of blood stasis rats. The related pathway and potential targets involved in the anti-BSS effects of Danshen were predicted by pathway analysis and network pharmacology. The biochemical results showed that Danshen intervention significantly reduced whole blood viscosity (WBV) at all the shear rates and fibrinogen concentration (FIB) (*p* < 0.01) and increased activated partial thromboplastin time (APTT) effectively (*p* < 0.01). We also found that 52 lipid metabolites, including glycerophospholipid, sphingolipid, glycerolipid, plasmalogen, cholesterol ester, and testosterone, were associated with blood stasis. Moreover, Dgka, Hsd17b3, Hsd3b1, Inppl1, Lpl, Pik3ca, Pik3r1, Pla2g1b, Pla2g2a, Soat1, and Soat2 were predicted as potential targets, while glycerophospholipid metabolism, glycerolipid metabolism, steroid and steroid hormone biosynthesis, phosphatidylinositol signaling system, and ether lipid metabolism were involved as shared critical pathways of lipidomics analysis and network pharmacology. Collectively, this study offered a new understanding of the protection mechanism of Danshen against BSS, which provided new insight to explore the protective effects of Danshen.

## 1. Introduction

Cardiovascular diseases (CVDs) are a leading cause of death for adults and remain a significant cause of health loss in all regions of the world [1, 2]. The earlier study has stated that 85.8% of 324 patients with CVDs also had blood stasis syndrome (BSS) [3]. Blood stasis syndrome, one of the most common traditional Chinese medicine (TCM) terminologies in cardiovascular diseases, is a significant symptom of patients with CVDs [4]. In TCM theory, blood that leaves the meridian, fails to be discharged or dissipated in time, and stays in a particular site is considered as blood stasis [5]. Modern medical researches reveal that pathological changes associated with blood stasis syndrome, including inflammation, thrombosis, and tissue edema, are caused by ischemia, hypoxia of tissue organs, blood viscosity abnormalities, and circulation disorders [6]. Hyperlipidemia is a widely recognized risk factor and plays an important role in CVDs generation and progression [7]. Literature also shows that, in the metabolomics analysis of rats with blood stasis, the fluctuations of endogenous metabolites may be responsible for this disease [8, 9]. But the underlying lipid metabolic mechanism and identification of potential lipid biomarkers remain equivocal.

Herbal medications are commonly applied for clinical applications, including treating CVDs, and have become more prominent in cardiovascular medicine among the various pharmaceutical prescriptions [10]. Besides, previous researches have reported that herbal medications had a significant curative effect on the treatment of blood stasis [11, 12]. Danshen, the dried root and rhizome of *Salvia miltiorrhiza*, is a versatile traditional Chinese herb widely utilized in China for improving body functions [13]. Danshen is believed to improve blood flow, promote coronary vasodilatation, prevent platelet aggregation, and suppress the formation of thromboxane [14]. Currently, there are over 900 commercial Danshen-based preparations available in China [15], such as Fufang Danshen Tablets and Fufang Danshen Dropping Pills, which have been extensively used for the prevention and amelioration of CVDs [16]. Although the curative ability and components of Danshen are well documented [17, 18], these studies still lack an interpretation of the intervention mechanism based on metabolomics/lipidomics technology.

Lipidomics, which was first introduced by Han and Gross [19], is a branch of metabolomics and a comprehensive understanding of all lipid species on the biological system [20]. With the rapid development of analytical techniques and methodologies, lipidomics provides a powerful tool to study disease states through alterations of lipid metabolites [21]. To discover the molecular mechanism of Danshen against BSS, the investigation of the complete biochemical analysis based on the untargeted lipidomic approach was thoroughly conducted in this study. As essential platforms for rapid interpretation of informative spectral datasets it can provide [22], multivariate analysis was employed to select the differential metabolites. Also, pathway analysis has become the first choice for assessing inherent biochemical reaction networks [23, 24], and network pharmacology based on systems biology emphasizes multichannel regulation of signaling pathways to improve the therapeutic effect of drugs [25]. Therefore, the related network between Danshen components and metabolic pathways can be constructed through pathway analysis and network pharmacology. Based on this, we can further understand the pathological mechanism of BSS and elaborate on the pharmacology mechanism of Danshen. However, neither lipidomics of BSS nor lipidomics of Danshen intervention on BSS has been studied.

In this work, an integrated approach of lipidomics and network pharmacology was performed to investigate the biological mechanisms of Danshen against BSS. Firstly, the metabolites' identification and metabolic pathway analysis were conducted through lipidomics based on a UPLC-QTOF-MS method. Secondly, the potential targets of Danshen treating BSS were predicted by network pharmacology. Finally, the shared pathways of lipidomics and network pharmacology were analyzed to discover the pharmacological mechanism of Danshen. This study interpreted the biological mechanisms of Danshen against BSS from the perspective of lipidomics and provided a new insight for the study of other diseases involved with disorders of lipid metabolism.

## 2. Methods

### 2.1. Chemicals and Materials

Danshen was purchased from The Chinese Medicine Material Processing Plant (Guangdong, China) and in accordance with the standard of Chinese Pharmacopoeia (2015 Edition). The herb was identified and authenticated in the Guangdong Pharmaceutical University. LC-MS grade acetonitrile, methanol, and isopropanol were attained from Merck KGaA (Darmstadt, Germany). HPLC grade MTBE and formic acid were purchased from TEDIA (Ohio, USA). Ammonium formate was purchased from Sigma-Aldrich (Darmstadt, Germany). The purified water was purchased from Watsons (Hong Kong, China). Aspirin was purchased from Zhourui Biotechnology Co., Ltd (Shanghai, China).

### 2.2. Danshen Decoction Preparation

Danshen was soaked in pure water (1 : 10, w/v) for 1 h at room temperature. The first reflux was performed for 2 h. The filtrate was then poured into a beaker, and the residue was recirculated with 8 volumes of pure water. The second reflux was performed for 2 h. Finally, all the filtrate was merged and evaporated to create 0.54 g/mL Danshen solution, which was then stored at −80°C for further usage.

### 2.3. Model Establishment

The rats were injected with 0.1% adrenaline hydrochloride (0.8 mg/kg) subcutaneously in the head and neck twice with an interval of 4 hours. After the first injection of adrenaline hydrochloride for 2 hours, the rats were subjected to ice water stimulation for 4 minutes to establish the acute blood stasis (ABS) rat models.

### 2.4. Animal Handling and Sample Collection

Thirty-two male Sprague-Dawley rats (200 ± 20 g) were provided by the Experimental Animal Center of Guangdong Province Traditional Chinese Medical Hospital (Guangzhou, China), certification SYXK 2013–0094, animal quality certificate No. 44002100018021. All of the rats were placed in the breeding room (temperature: 20 ± 3°C; humidity: 50 ± 5%) with free access to standard food and water, acclimated to the room for 7 days. The rats were monitored by the research team twice daily. The health of rats was monitored by food and water intake and general assessment of animal activity. All the animal experimental procedures were performed under the Guidelines of the Experimental Animal Center of Guangdong Province Traditional Chinese Medical Hospital and approved by the Animal Ethics Committee of Guangdong Province Traditional Chinese Medical Hospital. During the whole experiment, four investigators were responsible for the four different parts of this study (animal experiment, biochemical measurement, LC-MS analysis, and data statistical analysis).

All rats were included in this study and randomly divided into four groups (each *n* = 8) after acclimation for 7 days, namely, control group (CG), model group (MG), positive control group (PG), and Danshen-treated group (DG). The randomization was based on a computer-based random order generator. Rats in the positive control group were orally administered with aspirin at a dose of 0.05 mg/g; rats in the Danshen-treated group were orally administered with Danshen decoction at a dose of 1.35 mg/g; rats in both of control group and model group were given the equal dose of distilled water. The dose was given twice a day in the morning and evening, respectively, for 7 consecutive days. After the first administration on the seventh day, except the control group, the other groups were established to the ABS rat models according to the modeling method. All the groups were given the dose mentioned above after modeling. Then, blood was collected from the abdominal aorta for biochemical assay and LC-MS analysis after fasting 12 h under anesthesia with 3% isoflurane inhalation, which resulted in rats' death because of bleeding shock.

### 2.5. Measurement of Biochemical Parameters

The fibrinogen concentration (FIB), prothrombin time (PT), thrombin time (TT), and activated partial thromboplastin time (APTT) of the plasma were determined with C2000-4 blood coagulation instrument (Precil Instrument Co., Ltd, Beijing) and commercial determination kits. Whole blood viscosity (WBV) was determined with the LBY-N6K blood rheometer (Precil Instrument Co., Ltd, Beijing). All the measurements were conducted within a day.

### 2.6. Plasma Sample Preparation

300 *μ*L of cold MeOH was added to the 40 *μ*L plasma sample followed by 1 mL cold MTBE. Then, the mixture was vibrated at 120 rpm for 15 min at room temperature. After that, 300 *μ*L H_2_O was added and vortexed for 15 s to form a two-phase system. After equilibration for 10 min at 4°C, the mixture was centrifuged at 8000 rpm for 10 min at 4°C. The supernatant was lyophilized and redissolved in 150 *μ*L of ACN/IPA/H_2_O (65 : 30 : 5, v/v/v). The mixture was vortexed for 2 min and filtrated into the glass vial with inner lining-pipe through a 0.2 *μ*m filter membrane. The vials were stored at −80°C for further LC-MS analysis. An equal aliquot plasma of 32 rats was mixed to generate the pooled quality control (QC) sample, which was pretreated as above and used for method validation. It is noteworthy that the sample preparation process was strictly operated on the ice during the whole experiment to stabilize lipid molecules.

### 2.7. UPLC-MS Analysis

A 1290 Infinity UHPLC system coupled to Agilent 6530 QTOF mass spectrometer was used to analyze the lipid molecules. An Agilent Eclipse Plus C18 column (100 × 2.1 mm, 1.8 *μ*m) was used at a flow rate of 0.6 mL/min, and the column temperature was at 65°C. The sample chamber temperature was at 4°C. Each sample was analyzed twice with different LC gradients and mass spectrometer ionization modes to cover up both the positively charged and negatively charged species. For the positive mode, solvent A was 10 mM ammonium formate +0.1% formic acid in acetonitrile/water (60 : 40), and solvent B was 10 mM ammonium formate +0.1% formic acid in acetonitrile/isopropanol (10 : 90). The solvent gradient of the positive mode was as follows (v/v): 0–2 min, 15 to 30% B; 2–2.5 min, 30 to 48% B; 2.5–11 min, 48 to 82% B; 11–11.5 min, 82 to 99% B; 11.5–12 min, 99% B; 12–12.1 min, 99 to 15% B; 12.1–15 min, 15% B. For the negative mode, solvent A was 10 mM ammonium formate in acetonitrile/water (60 : 40), and solvent B was 10 mM ammonium formate in acetonitrile/isopropanol (10 : 90). The solvent gradient of the negative mode was as follows: 0–2 min, 15 to 30% B; 2–2.5 min, 30 to 48% B; 2.5–9.5 min, 48 to 76% B; 9.5–9.6 min, 76 to 99% B; 9.6–10.5 min, 99% B; 10.5–10.6 min, 99–15% B; 10.6–13.5 min, 15% B. Besides, the injection volumes between the two modes were different because of the discrepancy in response intensity under two opposite modes, which were 1 *μ*L for positive mode and 5 *μ*L for negative mode. System stability was examined by injecting a QC sample to every four specimens during the complete sample analysis. This analysis method can cover hundreds of lipids in one injection (Figure S1).

The optimized MS conditions were as follows: gas temperature 350°C, gas flow 10 L/min, nebulizer 30 psi, capillary voltage 3500 V (positive mode) and 3000 V (negative mode), skimmer voltage 65 V, octopole RF voltage 750 V, and fragmentor voltage 150 V. The full-scan mass range was 50–1500 Da. The targeted-MS/MS collision energy was set at 20 and 40 eV, respectively. A reference solution was sprayed as continuous calibration with the following reference masses: *m/z* 121.0509 and 922.0098 in positive mode and *m/z* 112.9856 and 1033.9881 in negative mode.

### 2.8. Data Processing and Analysis

Data obtained by QTOF were first converted into mzML format by ProteoWizard software and then processed in Progenesis QI v2.0 Software (Waters, Newcastle, UK). The optimal sample data was automatically selected by Progenesis QI to align all the remaining data. Subsequently, adduct ions were deconvoluted, and ion abundance above the threshold level was calculated. All features detected were matched and selected based on the Lipid Maps database within the mass tolerance of 10 ppm. The analyzed data was imported into Simca 14.1 (Umetrics, Umea, Sweden) for following multivariate statistical analysis such as orthogonal projections to latent structures-discriminate analysis (OPLS-DA) and principal component analysis (PCA). The ions with VIP values above 1.0 and *p* values of Student's *t*-test below 0.05 were selected as differential metabolites. These ions were further identified by tandem mass spectra.

### 2.9. Pathway Analysis and Network Pharmacology

The identified differential metabolites were imported into MetaboAnalyst for the pathway analysis. The visualization of the metabolite-pathway network was constructed by MetScape. The components of Danshen were collected from the Traditional Chinese Medicine Systems Pharmacology Database (https://tcmspw.com/index.php, TCMSP) and manually supplemented based on the literature [26]. To discover the targets of Danshen components, we used the Swiss Target Prediction Database (http://www.swisstargetprediction.ch/), which is a website for estimating the most probable macromolecular targets of a small molecule. Besides, the disease-related targets were searched from GeneCards Database (https://www.genecards.org/). Then, the shared targets of Danshen components and BSS were selected as potential targets for the KEGG pathway enrichment. Finally, the network containing relationships between components, potential targets, and metabolic pathways was established by the Cytoscape software.

### 2.10. Statistical Analysis

All the data are presented as the mean ± standard deviation (SD). Using SPSS Statistics 25.0, the statistical significance was determined by one-way ANOVA or Student's *t*-test. The significance level was set at *p* < 0.05 (significant) and *p* < 0.01 (extremely significant). Fold change was calculated to reflect the alteration levels of metabolites by dividing the mean of the metabolite intensity. Chemical similarity enrichment analysis (ChemRICH) was applied to perform chemical similarity-based statistical enrichment analysis. The heatmap analysis was performed by using PRISM GraphPad 8.0.

## 3. Results

### 3.1. Hemorheology and Coagulation Parameters

Whole blood viscosity, the main hemorheological parameter in this study, of rats in the four groups is shown in Table 1. Compared with the control group, all the shear rates of the WBV levels in the model group were significantly increased (*p* < 0.01), which indicated that the ABS rat models were successfully established. The WBV at all the shear rates was significantly reduced (*p* < 0.01) in the positive control group compared to the model group. In the Danshen-treated group, all the WBV indexes show similar declining trends as in the positive control group.

The effects of Danshen on blood coagulation function were measured by assessing of PT, APTT, FIB, and TT levels in the plasma. As shown in Table 2, the PT, APTT, and TT levels were significantly shortened, and the FIB level was significantly elevated in the model group compared to those in the control group (*p* < 0.01). The Danshen intervention was able to significantly increase APTT (*p* < 0.01), as well as lower FIB effectively (*p* < 0.01). After Danshen treatment, the PT and TT levels were increased to some extent but without any significant difference.

### 3.2. Reliability of the Lipidomics Platform

**Figure 1:**
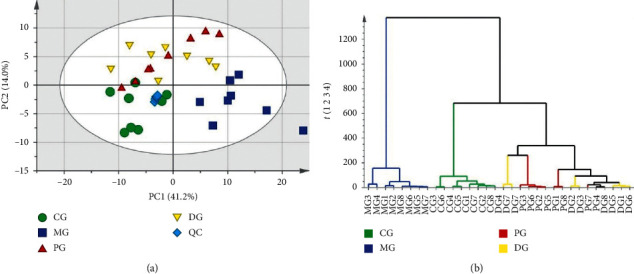
(a) PCA score plot (*n* = 8) of total differential metabolites in the positive and negative mode. (b) HCA plot of the CG, MG, PG, and DG groups was drawn based on the raw abundance of differential metabolites. CG: control group; MG: model group; PG: positive control group; DG: Danshen-treated group; QC: quality control.

High-quality data is essential in the lipidomics analysis. QC can determine whether the systematic error of the whole experiment is within the controllable range. In this study, PCA was applied to verify QC samples and estimate the repeatability and stability of this analytical method. From the PCA score plot (Figure 1(a)), the tightly clustered QC samples reflected the reliability of the UPLC-MS system and the high-quality of all the LC-MS data.

### 3.3. PCA and HCA for Lipidomics Analysis

PCA was used to exhibit the metabolic distinction among different groups for lipidomics. Using the PCA method, distinct changes in lipid metabolites patterns of rat plasma were observed. As illustrated in Figure 1(a), PC1 and PC2 accounted for 41.2% and 14.0% under both the positive and negative models. In the PCA score plot, a clear separation was displayed among the normal, blood stasis, and aspirin/Danshen-treated rats, indicating that metabolic perturbations occurred in rats depending on pathological conditions and pharmacological intervention. Besides, most Danshen-treated samples were closer to the control group and shown a regressive trend from the model group to the control group, suggesting the potential curative effect of Danshen on blood stasis. Hierarchical cluster analysis (HCA) was computed with Ward's linkage algorithm and sorted by size. HCA plot (Figure 1(b)) showed that the above four groups could be classified clearly into three groups: group I (model group), group II (control group), and group III (positive group +Danshen group). Besides, the model group showed a clear difference with the other three experimental groups in the HCA plot. Therefore, the above results of the HCA plot were consistent with the results of the PCA analysis.

### 3.4. Blood Stasis Affected Lipid Metabolism in Rats

**Figure 2:**
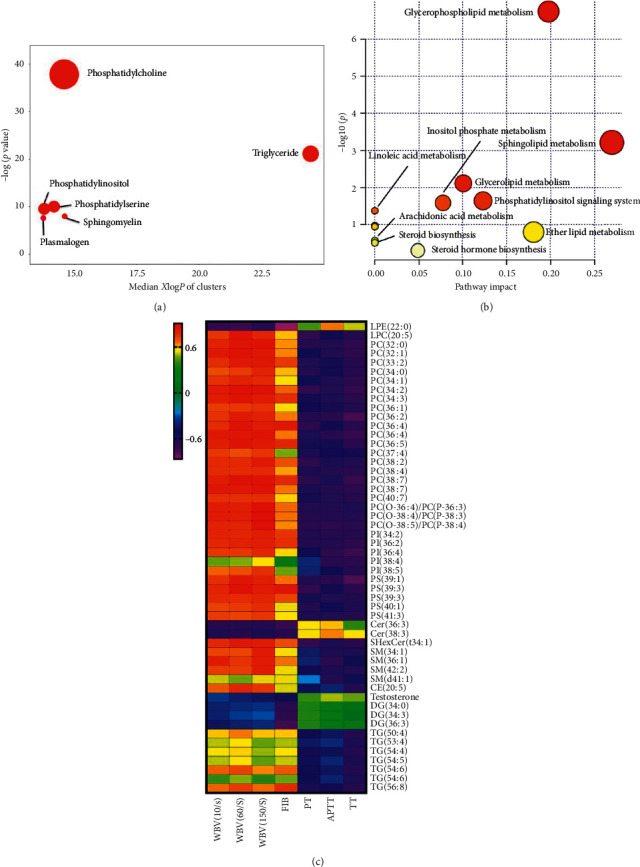
(a) Chemical similarity enrichment analysis plot. (b) Heatmap of Spearman's correlation between differential metabolites and biochemical parameters. (c) Summary plot of metabolic pathways associated with differential metabolites.

A total of 52 metabolites was identified from lipidomics analysis (Table S1). All the identified metabolites met the screening criteria (VIP > 1.0 and *p* < 0.05), and the metabolic features were matched by comparing the MS/MS fragments within online databases, such as HMDB and Lipid Blast. From the result of ChemRICH, 6 significantly changed clusters were found more related to the risk of BBS (Figure 2(a), Table S2), including phosphatidylcholines (PCs), triglycerides (TGs), phosphatidylinositols (PIs), phosphatidylserines (PSs), sphingomyelins (SMs), and plasmalogens. For further statistical analysis, 45 metabolites were found significantly increased, and the remaining 7 metabolites were significantly decreased in the model group compared with the control group. Specifically, LPC (20 : 5), PCs, plasmalogens, PIs, PSs, SHexCer (t34 : 1), SMs, 20 : 5 cholesterol ester (CE (20 : 5)), and TGs were significantly increased in the model group, whereas the concentrations of LPE (22 : 0), Ceramides (Cers), Diacylglycerols (DGs), and testosterone were significantly decreased compared with the control group.

To explore the connection between the lipid metabolites and biochemical parameters, the Spearman correlation test was performed. The correlations were visualized through a heatmap (Figure 2(b)). In total, 52 differential metabolites were correlated with at least one of the BSS-related physiological traits. The values of |*r*| > 0.6 were taken as strong correlations (*r* represents correlation coefficient). Among these 52 metabolites, we observed strong correlations between metabolites and WBV indexes than coagulation parameters. Noteworthily, the FIB parameter showed similar correlations with metabolites as WBV indexes, which was contrary to the other coagulation parameters. LPC (20 : 5), PCs, plasmalogens, PIs, PSs, SHexCer (t34 : 1), SMs, CE (20 : 5), and TGs showed positive correlations with WBVs and FIB and negative correlations with PT, APTT, and TT. Also, LPE (22 : 0), Cers, testosterone, and DGs were negatively associated with WBVs and FIB and positively correlated with the other three coagulation parameters.

**Figure 3:**
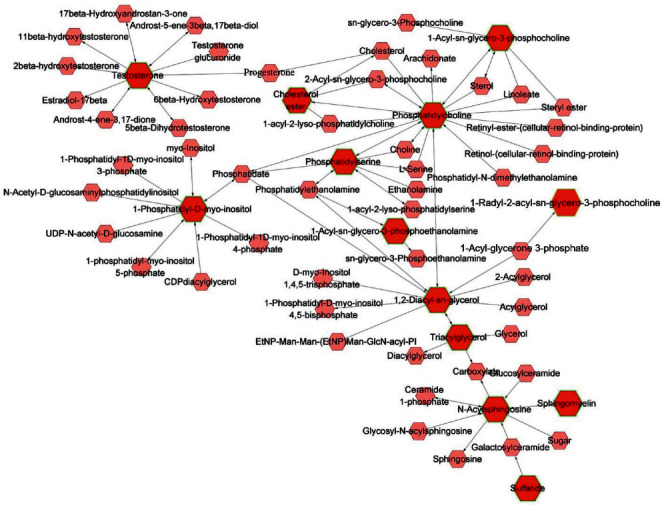
The network of the remarkably perturbed metabolic pathways in BSS by MetScape analysis. The red hexagons represented the identified differential lipid metabolites, and the pink ones were the involved metabolites that have not been identified in this study.

To better understand the biological function of these altered lipid metabolites, matched metabolic pathways were calculated based on the pathway impact values and *p* values through KEGG pathway enrichment and topology analysis. A total of 12 pathways were found related to this disease, and 6 of them were significantly influenced by BSS (*p* < 0.05, Figure 2(c), Table S3) including glycerophospholipid metabolism, sphingolipid metabolism, glycerolipid metabolism, phosphatidylinositol signaling system, and linoleic acid metabolism. The detailed pathway-based network based on these remarkably perturbed metabolites was constructed by MetScape (Figure 3).

### 3.5. Effect of Danshen Extract on Blood Stasis

**Figure 4:**
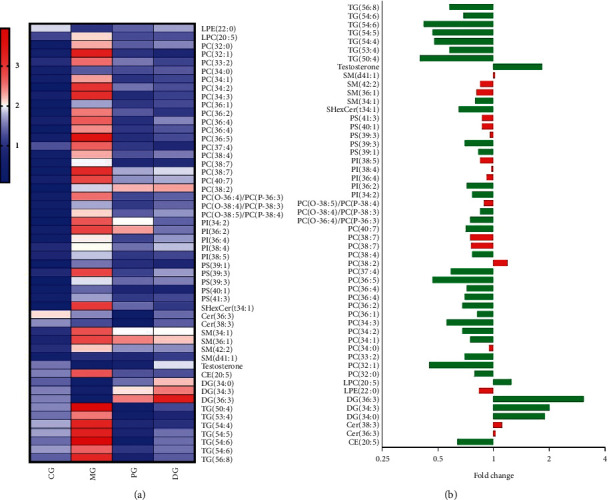
(a) Heatmap based on the normalized raw abundance of differential metabolites. (b) Changed metabolites between the model group and the Danshen-treated group: green means significant difference (*p* < 0.05), and red means no significant difference (*p* > 0.05). CG: control group; MG: model group; PG: positive control group; DG: Danshen-treated group.

As presented in Figure 1(a), the Danshen-treated group was located between the control group and the model group in the PCA score plot and overlapped with the positive group, suggesting that Danshen could ameliorate the dysfunction of lipid metabolism related to this disease. To further understand the specific metabolic changes, a heatmap of differential lipids was conducted, and the normalized concentration of each metabolite was calculated based on the raw abundance (Figure 4(a)). Compared with the control group, LPC (20 : 5), PCs, plasmalogens, PIs, PSs, SHexCer (t34 : 1), CE (20 : 5), SMs, and TGs exhibited higher contents in the model group, whereas Cers, DGs, LPE (22 : 0), and testosterone showed lower contents in the model group. After the treatment of Danshen, it was observed that concentrations of 35 differential lipids in the model group were significantly restored (*p* > 0.05). In general, Danshen intervention could improve abnormal lipid metabolism. Nevertheless, Danshen had little influence on the Cers and SMs, as shown in Figure 4(b).

### 3.6. Network Pharmacology

**Figure 5:**
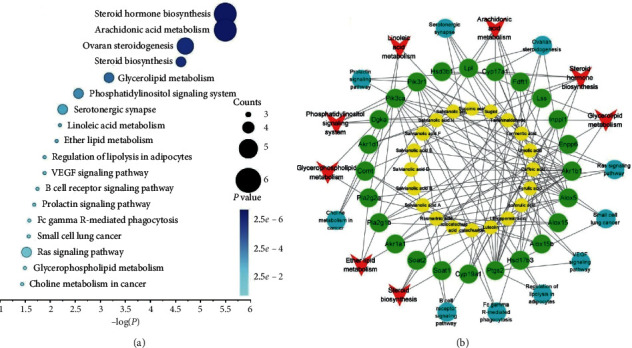
(a) KEGG enrichment scatter plot of targets related to Danshen components. (b) The component-target-pathway network (yellow: compounds, green: predicted targets, blue: irrelevant metabolic pathways, red: pathways related to lipidomics analysis).

Conjointly, the above lipidomics data provided compelling evidence of a preventive effect of Danshen in the blood stasis model rats. As a well-known Chinese herb, Danshen also has the characteristics of multiple components related to diverse targets and metabolic pathways. Thus, network pharmacology technology was applied to predict component-related targets and obtain potential metabolic pathways, which established the foundation for further intervention mechanism studies. Based on the preceding databases and literature, we collected 20 typical Danshen components and 24 disease-related genes as potential drug-targets (Table S4). These potential targets were analyzed and predicted by the KEGG pathway database, and 18 related metabolic pathways (*p* < 0.05) were obtained, as shown in Figure 5(a) and Table S5. To visually reveal the relationships among the components, targets, and metabolic pathways, the component-target-pathway interaction network was constructed by the Cytoscape software (Figure 5(b)). Among all the predicted pathways, 8 pathways represented as red circles in Figure 5(b) were shared with previous pathways of lipidomics analysis. These pathways were considered as critical pathways to interpret the mechanism of Danshen intervention.

## 4. Discussion

### 4.1. Biochemistry Analysis

Biochemical parameters have been extensively applied for the diagnosis of BSS. The WBV indexes and coagulation parameters, including APTT, TT, PT, and FIB, were employed to estimate the effectiveness of the antithrombotic activity of Danshen. Aspirin, a common drug that inhibits platelet aggregation, was used as the positive control drug in this study. Blood stasis can interfere with normal blood flow, which causes hemorheological abnormalities. WBV is the reflection of the internal resistance of blood flow in vessels [27]. In this study, WBV significantly increased at all shear rates in the blood stasis rats, which meant the reduction of the blood fluidity. The Danshen treatment could significantly decrease WBV at all shear rates, indicating that Danshen could reduce blood flow resistance and promote blood circulation. This research further detected the influence on plasma coagulation parameters, reflecting pathological changes in the coagulation system. PT and APTT are used to evaluate the overall efficiency of extrinsic and intrinsic clotting activities, respectively. TT and FIB are related to the common coagulation pathway in plasma, and the increased TT or the reduction of FIB indicates inhibition of thrombin-mediated fibrin formation [28]. After Danshen administration, the APTT, TT, PT, and FIB levels partially restored to the normal values, indicating that Danshen regulates the abnormality of the coagulation system and alleviates acute blood stasis.

### 4.2. Lipid Metabolism

In this study, lipidomics analysis revealed that blood stasis mainly altered the levels of glycerophospholipids, sphingolipids, glycerolipids, plasmalogens, CE (20 : 5), testosterone, and their corresponding metabolic pathways in the model rats. According to the result of network pharmacology, the multicomponents of Danshen regulated multiple targets through related metabolic pathways to ameliorate blood stasis in model rats. After screening irrelevant targets and pathways, the involved metabolic pathways included glycerophospholipid metabolism, sphingolipid metabolism, glycerolipid metabolism, steroid and steroid hormone biosynthesis, phosphatidylinositol signaling system, and ether lipid metabolism.

#### 4.2.1. Glycerophospholipid Metabolism

The most significant alteration of lipids in the present study was the increased levels of glycerophospholipid in blood stasis rats. PCs are the most abundant phospholipid in mammalian cell types and a major source of choline and trimethylamine, which are metabolized into trimethylamine oxide (TMAO). Compelling evidence suggests that high circulating TMAO concentration has been related to an increased risk of CVDs and mortality [29]. PCs also serve as a direct substrate for arachidonic acid and linoleic acid synthesis in the body. The significantly increased levels of PCs in the model rats suggested an imbalance in the arachidonic acid and linoleic acid metabolism. The metabolites in these two metabolic pathways contributed to platelet aggregation and inflammation, which are closely related to blood stasis [30]. LPC and LPE are products of PC and PE, respectively, which are structural components of animal cell membranes. Almost 50% of PC in low-density lipoprotein (LDL) particles are converted into LPCs [31]. LPCs, the main component of OxLDL, induce remarkable proinflammatory effects and reduce vascular relaxation [32]. Therefore, the increased level of LPC (20 : 5) played an important role in damaging vessels, inducing inflammation, and blocking blood circulation.

PS provides another attractive target for the pathogenic mechanism of blood stasis being a natural phospholipid with multiple anti-inflammation functions, which is also widely accepted to act as a key regulator of hemostasis and coagulation [33, 34]. In this study, the increased levels of PSs enhanced the effect of hemostasis and coagulation. The functions of hemostasis and coagulation are closely related to blood circulation disorders, which lead to the formation of blood stasis. During hemostasis and coagulation, PS plays a role in maintaining a balance between pro- and anticoagulant activities.

The predicted targets involved in this metabolic pathway were Pla2g1b and Pla2g2a, which belonged to the family of secretory phospholipases A_2_ (sPLA_2_). The sPLA_2_ family is responsible for phospholipid hydrolysis at the sn-2 position to produce multiple potentially bioactive lipids [35, 36]. Our results showed that the upregulated glycerophospholipids could be significantly restored after Danshen treatment, indicating that Danshen components might activate relative phospholipases to resolve the disturbance of glycerophospholipid metabolism.

#### 4.2.2. Sphingolipid Metabolism

Sphingolipids are bioactive components of cell membranes, which play a crucial part in cell growth, differentiation, and apoptosis. Sphingolipid and sphingolipid metabolism have already been implicated in the pathogenesis of CVDs [37]. Cer and SM are the two main species in sphingolipid metabolism. Several enzymatic pathways in mammalian cells generate Cers. Cer has been shown to regulate vascular tone or vasomotor responses in various vascular beds [38]. The decreased concentrations of Cers inhibit vasodilator or vasoconstrictor effects. SM can be generated from ceramide by sphingomyelin synthase. According to the reported literature [39], the plasma SM was in the biological pathways that mediate the risk for subclinical disease attributable to CVD risk factors. Sulfatide is synthesized by two transferases from Cer and is degraded explicitly by a sulfatase. Sulfatide enhances thrombosis and accelerates coagulation, possibly through the participation of blood coagulation factor XII and binding with annexin V, respectively. Also, the interaction between P-selectin and sulfatide is vital for stable platelet adhesion and aggregation [40]. Because of no potential targets related to Danshen components, only SHexCer (t34 : 1) returned to the normal level with remarkable change after the treatment of Danshen, whereas Cers and SMs showed no significant effect of Danshen extract.

#### 4.2.3. Glycerolipid Metabolism

The lipidomics results indicated that TG and DG involved in glycerolipid metabolism showed significant perturbations in the model rats. Medium and high concentrations of TGs have been regarded as a cardiovascular risk factor for more than 30 years [41]. The accumulation of TGs, particularly in nonadipose tissues, is an essential reflection of lipotoxicity [21]. TG species can be reacylated from corresponding DG species by diglyceride acyltransferase. Lipoprotein lipase (Lpl) is a multifunctional protein involved in glycerolipid metabolism. Lpl hydrolyses core TG from TG-rich particles to form remnant lipoproteins and regulates the deacetylation of TGs [42]. The deficiency of Lpl function leads to the accumulation of TGs, which is the possible cause of blood stasis. Diacylglycerol kinase (Dgka), another potential target, catalyzes the direct phosphorylation of DG to form phosphatidic acid [43]. From the metabolic results, the dysfunction of this metabolic pathway was recovered with Danshen intervention. The Danshen treatment might activate Lpl and inhibit Dgka to restore the concentrations of TGs and DGs.

#### 4.2.4. Steroid and Steroid Hormone Biosynthesis

Testosterone, a kind of sex hormone, has multiple influences on the cardiovascular system. The decreased level of testosterone is associated with the production of proinflammatory cytokines and increased arterial thickness and total cholesterol [44]. Testosterone also has relaxation effects on the blood vessels to promote blood circulation [45]. Therefore, the declined concentration of testosterone is an important cause leading to the formation of blood stasis. Testosterone synthesis is regulated by multiple enzymes, including 17*β*-hydroxysteroid dehydrogenases (Hsd17b) and 3*β*-hydroxysteroid dehydrogenases (Hsd3b) [46, 47]. These targets are represented as Hsd17b3 and Hsd3b1 in this study. Danshen treatment might upregulate the concentration of testosterone by regulating relative enzymes to balance the synthesis process.

Cholesterol ester, a major component of atherogenic lipoproteins, emerged to play a critical role in the development of atherosclerosis [48]. The enzyme sterol O-acyltransferases (Soat1 and Soat2), which catalyze the synthesis of CE from free cholesterol, are regarded as a potential target for atherosclerosis and hypercholesterolemia [49, 50]. In the blood stasis rats, the significant accumulation of CE (20 : 5) might contribute to the overactivation of Soat enzymes. However, as the potential targets, the overactivated Soat enzymes might be inhibited by Danshen components to function normally according to the lipidomics results.

#### 4.2.5. Phosphatidylinositol Signaling System

PI, a vital component in the cell membrane, plays a pivotal role in cell morphology, metabolic regulation, and signal transduction. The potential targets involved in this pathway were Pik3ca and Pik3r1, which belonged to the family of phosphatidylinositol 3-kinase (Pi3k). As a vital phosphokinase, Pi3k can specifically catalyze PIs and their derivatives to produce phosphatidylinositol 3,4,5-triphosphate (PIP3) [51]. Pi3k activation enhances cell survival and antagonizes apoptosis in many cell types, including cardiomyocytes, cardiac fibroblasts, and endothelial cells [52]. Pi3k also regulates the secretion of vascular endothelial growth factors and migration of vascular endothelial cells to promote the process of angiogenesis [53]. The regenerated blood vessels form the collateral circulation to partly enhance blood circulation in the body. Inositol polyphosphate phosphatase-like 1(Inppl1) acts downstream of Pi3k to dephosphorylate PIP3. Irregular expression of Inppl1 may disrupt the normal signaling pathway [54]. Therefore, the accumulation of PIs indicated the suppression of Pi3k and Inppl1 activation and contributed to the formation of blood stasis. The restored concentrations of PTs might be attributed to the reactivation of Pi3k and Inppl1 regulated by the Danshen intervention.

#### 4.2.6. Ether Lipid Metabolism

Plasmalogen, also known as a subclass of vinyl ether-containing glycerophospholipid, has many roles in cellular function [55]. The presence of vinyl ether bonds makes plasmalogens more vulnerable to oxidative attack compared to their corresponding 1-acyl analogs [56, 57]. Plasmalogens are consumed in this oxidation reaction as sacrificial oxidants to protect other lipids from being oxidized. These lipid oxidation products have been identified to have diverse and potent effects on inflammatory and reparative responses [58]. The perturbations of plasmalogens may cause an imbalance of process in lipid oxidation. The relevant targets in this pathway were Pla2g2a and Pla2g1b, which have been introduced in glycerophospholipid metabolism. Phospholipase A_2_ catalyzes the hydrolysis of the sn-2 fatty acyl bond of plasmalogens to liberate free fatty acids and lysophospholipids. The related components might activate sPla_2_ enzymes to reduce the accumulation of plasmalogens in the blood stasis rats.

## 5. Conclusion

In the current work, lipidomics analysis based on UHPLC-QTOF-MS technology showed that 52 differential lipids were related to BSS, including glycerophospholipids, sphingolipids, glycerolipids, plasmalogens, CE (20 : 5), and testosterone. Integrating with pathway analysis and network pharmacology, Dgka, Hsd17b3, Hsd3b1, Inppl1, Lpl, Pik3ca, Pik3r1, Pla2g1b, Pla2g2a, Soat1, and Soat2 were predicted as potential targets of Danshen treating BSS. In summary, this study preliminarily elucidated the pathological mechanism of BSS and the pharmacological mechanism of Danshen against BSS, which provided new insight to explore the protective effects of Danshen.

## Figures and Tables

**Table 1:** Trends of WBV index of rats in each group.

Group	WBV (mPa·s)		
	10 (s)	60 (s)	150 (s)
CG	8.68 ± 0.93	5.19 ± 0.32	4.02 ± 0.15
MG	13.75 ± 0.72^##^	6.80 ± 0.13^##^	5.10 ± 0.20^##^
PG	10.79 ± 1.10 ^*∗∗*^	5.94 ± 0.48 ^*∗∗*^	4.54 ± 0.30 ^*∗∗*^
DG	9.89 ± 1.77 ^*∗∗*^	5.68 ± 0.58 ^*∗∗*^	4.63 ± 0.48 ^*∗*^

CG: control group; MG: model group; PG: positive control group; DG: Danshen-treated group. Data represent mean ± SD, *n* = 8. ^#^*p* < 0.05 and ^##^*p* < 0.01, compared with CG;  ^*∗*^*p* < 0.05 and  ^*∗∗*^*p* < 0.01, compared with MG.

**Table 2:** Coagulation parameters of rats in each group.

Group	PT (s)	APTT (s)	FIB (g/L)	TT (s)
CG	14.15 ± 1.82	20.19 ± 3.27	1.77 ± 0.19	34.51 ± 1.66
MG	12.15 ± 0.52^##^	13.95 ± 2.09^##^	3.21 ± 0.12^##^	28.70 ± 4.90^##^
PG	13.15 ± 0.80 ^*∗*^	18.32 ± 1.57 ^*∗∗*^	3.03 ± 0.13 ^*∗*^	32.10 ± 3.63
DG	12.70 ± 1.06	17.75 ± 2.09 ^*∗∗*^	3.04 ± 0.08 ^*∗∗*^	30.65 ± 2.98

CG: control group; MG: model group; PG: positive control group; DG: Danshen-treated group. Data represent mean ± SD, *n* = 8. ^#^*p* < 0.05 and ^##^*p* < 0.01, compared with CG;  ^*∗*^*p* < 0.05 and  ^*∗∗*^*p* < 0.01, compared with MG.

## Data Availability

The data used and analyzed in this study are available from the corresponding author on reasonable request.

## Abbreviations

LC-MS: Liquid chromatography-mass spectrometry
ESI: Electrospray ionization
CVD: Cardiovascular diseases
BBS: Blood stasis syndrome
UPLC-QTOF-MS: Ultra-High-Performance Liquid Chromatography-Quadrupole-Time-of-Flight-Mass Spectrometry
LDL: Low-density lipoprotein
Cer: Ceramide
TT: Thrombin time
PT: Prothrombin time
APTT: Activated partial thromboplastin time
FIB: Fibrinogen concentration
WBV: Whole blood viscosity
MTBE: Methyl tert-butyl ether
ACN: Acetonitrile
IPA: Isopropyl alcohol
QC: Quality control
FC: Fold change
OPLS-DA: Orthogonal partial least squares discrimination Analysis
SD: Standard deviation
LPC: Lysophosphatidylcholine
LPE: Lysophosphatidylethanolamine
PC: Phosphatidylcholine
SM: Sphingomyelin
SHexCer: (3'-sulfo) Galbeta-Ceramide
PC(O/P): Plasmalogen
DG: Diglyceride
TG: Triglyceride
CE: Cholesteryl ester
PI: Phosphatidylinositol
PS: Phosphatidylserine
PCA: Principal component analysis
PC: Principal component
HCA: Hierarchical clustering analysis.

## Supplementary Materials

Supplementary Materials Figure S1: (a) LC-MS positive TIC diagram of rats plasma; (b) LC-MS negative TIC diagram of rats plasma. Table S1: MS-based identification of the lipid molecular species detected in the present study. Table S2: chemical similarity enrichment analysis of differential metabolites. Table S3: disturbed metabolic pathways in BSS. Table S4: the typical components and related targets in *Salvia miltiorrhiza* (Danshen). Table S5: functional enrichment result of potential targets.

## References

[1] Roth G. A., Johnson C., Abajobir A. (2017). Global, regional, and national burden of cardiovascular diseases for 10 causes, 1990 to 2015. *Journal of the American College of Cardiology*.

[2] Clark H. (2013). NCDs: a challenge to sustainable human development. *The Lancet*.

[3] Wang J., Chu F.-Y., Li J. (2008). Study on syndrome element characteristics and its correlation with coronary angiography in 324 patients with coronary heart disease. *Chinese Journal of Integrative Medicine*.

[4] Jang S., Ko M. M., Kang B. K., Jung J. (2020). Perception of metabolic diseases related to blood stasis: a survey of Korean medicine doctors. *European Journal of Integrative Medicine*.

[5] Wang Y., Li C., Chang H. (2016). Metabolomic profiling reveals distinct patterns of tricarboxylic acid disorders in blood stasis syndrome associated with coronary heart disease. *Chinese Journal of Integrative Medicine*.

[6] Chen K.-J. (2012). Blood stasis syndrome and its treatment with activating blood circulation to remove blood stasis therapy. *Chinese Journal of Integrative Medicine*.

[7] Nelson R. H. (2013). Hyperlipidemia as a risk factor for cardiovascular disease. *Primary Care: Clinics in Office Practice*.

[8] Han Y., Li Y., Wang Y., Gao J., Xia L., Hong Y. (2016). Comparison of fresh, dried and stir-frying gingers in decoction with blood stasis syndrome in rats based on a GC-TOF/MS metabolomics approach. *Journal of Pharmaceutical and Biomedical Analysis*.

[9] Wu J.-X., Zheng H., Yao X. (2019). Comparative analysis of the compatibility effects of Danggui-Sini Decoction on a blood stasis syndrome rat model using untargeted metabolomics. *Journal of Chromatography B*.

[10] Liperoti R., Vetrano D. L., Bernabei R., Onder G. (2017). Herbal medications in cardiovascular medicine. *Journal of the American College of Cardiology*.

[11] Huang H., Wu J., Lu R. (2020). Dynamic urinary metabolomics analysis based on UHPLC-Q-TOF/MS to investigate the potential biomarkers of blood stasis syndrome and the effects of Danggui Sini decoction. *Journal of Pharmaceutical and Biomedical Analysis*.

[12] Cao D., Xu C., Xue Y. (2018). The therapeutic effect of Ilex pubescens extract on blood stasis model rats according to serum metabolomics. *Journal of Ethnopharmacology*.

[13] Meim X.-D., Cao Y.-F., Che Y.-Y. (2019). Danshen: a phytochemical and pharmacological overview. *Chinese Journal of Natural Medicines*.

[14] Cheng T. O. (2007). Cardiovascular effects of danshen. *International Journal of Cardiology*.

[15] Wang L., Venitz J., Sweet D. H. (2014). Cumulative organic anion transporter-mediated drug-drug interaction potential of multiple components in salvia miltiorrhiza (Danshen) preparations. *Pharmaceutical Research*.

[16] Li M.-H., Chen J.-M., Peng Y., Wu Q., Xiao P.-G. (2008). Investigation of Danshen and related medicinal plants in China. *Journal of Ethnopharmacology*.

[17] Zhou L., Zuo Z., Chow M. S. S. (2005). Danshen: an overview of its chemistry, pharmacology, pharmacokinetics, and clinical use. *The Journal of Clinical Pharmacology*.

[18] Li Z.-M., Xu S.-W., Liu P.-Q. (2018). Salvia miltiorrhizaBurge (Danshen): a golden herbal medicine in cardiovascular therapeutics. *Acta Pharmacologica Sinica*.

[19] Han X., Gross R. W. (2003). Global analyses of cellular lipidomes directly from crude extracts of biological samples by ESI mass spectrometry: a bridge to lipidomics. *Journal of Lipid Research*.

[20] Watson A. D. (2006). Thematic review series: systems biology approaches to metabolic and cardiovascular disorders. lipidomics: a global approach to lipid analysis in biological systems. *Journal of Lipid Research*.

[21] Han X. (2016). Lipidomics for studying metabolism. *Nature Reviews Endocrinology*.

[22] Worley B., Powers R. (2013). Multivariate analysis in metabolomics. *Current Metabolomics*.

[23] Klamt S., Stelling J. (2003). Two approaches for metabolic pathway analysis?. *TRENDS in Biotechnology*.

[24] Khatri P., Sirota M., Butte A. J. (2012). Ten years of pathway analysis: current approaches and outstanding challenges. *PLoS Computational Biology*.

[25] Hopkins A. L. (2008). Network pharmacology: the next paradigm in drug discovery. *Nature Chemical Biology*.

[26] Zhao Q., Song Z., Fang X. (2016). Effect of genotype and environment on salvia miltiorrhiza roots using LC/MS-Based metabolomics. *Molecules*.

[27] Liu L., Duan J.-A., Tang Y. (2012). Taoren-Honghua herb pair and its main components promoting blood circulation through influencing on hemorheology, plasma coagulation and platelet aggregation. *Journal of Ethnopharmacology*.

[28] Dang X., Miao J.-J., Chen A.-Q. (2015). The antithrombotic effect of RSNK in blood-stasis model rats. *Journal of Ethnopharmacology*.

[29] Zheng Y., Li Y., Rimm E. B. (2016). Dietary phosphatidylcholine and risk of all-cause and cardiovascular-specific mortality among US women and men. *The American Journal of Clinical Nutrition*.

[30] Shen D., Ma N., Yang Y. (2019). UPLC-Q-TOF/MS-Based plasma metabolomics to evaluate the effects of aspirin eugenol ester on blood stasis in rats. *Molecules*.

[31] Park J. Y., Lee S. H., Shin M. J., Hwang G. S. (2015). Alteration in metabolic signature and lipid metabolism in patients with angina pectoris and myocardial infarction. *PLoS One*.

[32] Liu P., Zhu W., Chen C. (2020). The mechanisms of lysophosphatidylcholine in the development of diseases. *Life Sciences*.

[33] Darabi M., Kontush A. (2016). Phosphatidylserine in atherosclerosis. *Current Opinion in Lipidology*.

[34] Lentz B. R. (2003). Exposure of platelet membrane phosphatidylserine regulates blood coagulation. *Progress in Lipid Research*.

[35] Rosenson R. S. (2009). Future role for selective phospholipase A2 inhibitors in the prevention of atherosclerotic cardiovascular disease. *Cardiovascular Drugs and Therapy*.

[36] Rosenson R. S., Hurt-Camejo E. (2012). Phospholipase A2 enzymes and the risk of atherosclerosis. *European Heart Journal*.

[37] Kurz J., Parnham M. J., Geisslinger G., Schiffmann S. (2019). Ceramides as novel disease biomarkers. *Trends in Molecular Medicine*.

[38] Li X., Becker K. A., Zhang Y. (2010). Ceramide in redox signaling and cardiovascular diseases. *Cellular Physiology and Biochemistry*.

[39] Nelson J. C., Jiang X.-C., Tabas I., Tall A., Shea S. (2006). Plasma sphingomyelin and subclinical atherosclerosis: findings from the multi-ethnic study of atherosclerosis. *American Journal of Epidemiology*.

[40] Takahashi T., Suzuki T. (2012). Role of sulfatide in normal and pathological cells and tissues. *Journal of Lipid Research*.

[41] Nordestgaard B. G., Varbo A. (2014). Triglycerides and cardiovascular disease. *The Lancet*.

[42] Hegele R. A. (2016). Multidimensional regulation of lipoprotein lipase: impact on biochemical and cardiovascular phenotypes. *Journal of Lipid Research*.

[43] Horn W. D. V., Kim H. J., Ellis C. D. (2009). Solution nuclear magnetic resonance structure of membrane-integral diacylglycerol kinase. *Science*.

[44] Bianchi V. E. (2018). Testosterone, myocardial function, and mortality. *Heart Failure Reviews*.

[45] Oskui P. M., French W. J., Herring M. J., Mayeda G. S., Burstein S., Kloner R. A. (2013). Testosterone and the cardiovascular system: a comprehensive review of the clinical literature. *Journal of the American Heart Association*.

[46] Mindnich R., Moller G., Adamski J. (2004). The role of 17 beta-hydroxysteroid dehydrogenases. *Molecular and Cellular Endocrinology*.

[47] Ye L., Su Z.-J., Ge R.-S. (2011). Inhibitors of testosterone biosynthetic and metabolic activation enzymes. *Molecules*.

[48] Zhang J., Sawyer J. K., Marshall S. M. (2014). Cholesterol esters (CE) derived from hepatic sterol O-acyltransferase 2 (SOAT2) are associated with more atherosclerosis than CE from intestinal SOAT2. *Circulation Research*.

[49] Ohshiro T., Kobayashi K., Ohba M. (2017). Selective inhibition of sterolO-acyltransferase 1 isozyme by beauveriolide III in intact cells. *Scientific Reports*.

[50] Guan C., Niu Y., Chen S. C. (2020). Structural insights into the inhibition mechanism of human sterol O-acyltransferase 1 by a competitive inhibitor. *Nature Communications*.

[51] Kapeller R., Cantley L. C. (1994). Phosphatidylinositol 3-kinase. *BioEssays*.

[52] Oudit G., Sun H., Kerfant B. G. (2004). The role of phosphoinositide-3 kinase and PTEN in cardiovascular physiology and disease. *Journal of Molecular and Cellular Cardiology*.

[53] Namkoong S., Kim C.-K., Cho Y.-L. (2009). Forskolin increases angiogenesis through the coordinated cross-talk of PKA-dependent VEGF expression and Epac-mediated PI3K/Akt/eNOS signaling. *Cellular Signalling*.

[54] Fu M., Gu X., Ni H. (2013). High expression of inositol polyphosphate phosphatase-like 1 associates with unfavorable survival in hepatocellular carcinoma. *International journal of clinical and experimental pathology*.

[55] Yang K., Zhao Z., Gross R. W., Han X. (2007). Shotgun lipidomics identifies a paired rule for the presence of isomeric ether phospholipid molecular species. *PLoS One*.

[56] Brites P., Waterham H. R., Wanders R. J. (2004). Functions and biosynthesis of plasmalogens in health and disease. *Biochimica et Biophysica Acta*.

[57] Paul S., Lancaster G. I., Meikle P. J. (2019). Plasmalogens: a potential therapeutic target for neurodegenerative and cardiometabolic disease. *Progress in Lipid Research*.

[58] McIntyre T. M., Hazen S. L. (2010). Lipid oxidation and cardiovascular disease: introduction to a review series. *Circulation Research*.

