# Supplementary material for: Combined Lipidomics and Network Pharmacology Study of Protective Effects of Salvia miltiorrhiza against Blood Stasis Syndrome
Source: Evid Based Complement Alternat Med. 2021 Mar 19;2021:5526778. doi: 10.1155/2021/5526778 (PMC7997765; doi:10.1155/2021/5526778)
Supplement: Supplementary Materials — Figure S1: (a) LC-MS positive TIC diagram of rats plasma; (b) LC-MS negative TIC diagram of rats plasma. Table S1: MS-based identification of the lipid molecular species detected in the present study. Table S2: chemical similarity enrichment analysis of differential metabolites. Table S3: disturbed metabolic pathways in BSS. Table S4: the typical components and related targets in Salvia miltiorrhiza (Danshen). Table S5: functional enrichment result of potential targets. [file 5526778.f1.docx]

**Supplementary information**

**Combined lipidomics and network pharmacology study of protective effects of *Salvia miltiorrhiza* against blood stasis syndrome**

Yidian Jin^a1^, Zhiru Xie^b,d1^, Shasha Li^b^, Xiangyu Zeng^a^, Leqi Wang^b,d^, Ping Hu^a^,

Hongyang Zhang^a,c*^, Xue Xiao^d*^

^a^ School of Chemistry and Molecular Engineering, East China University of Science and Technology, Shanghai 200237, China

^b^ The Second Affiliated Hospital of Guangzhou University of Chinese Medicine, Guangzhou, 510120, China

^c^ Shanghai Key Laboratory of New Drug Design, School of Pharmacy, East China University of Science and Technology, Shanghai 200237, China

^d^ Institute of Traditional Chinese Medicine, Guangdong Pharmaceutical University, Guangzhou 510006, China

^1^ These authors contributed equally to this work.

**^*^** Corresponding author. Tel.: +86-21-64252844; +86-20-39353115.

E-mail: hongyang_zhang@ecust.edu.cn (H. Zhang); erxiaohappy@163.com (X. Xiao).


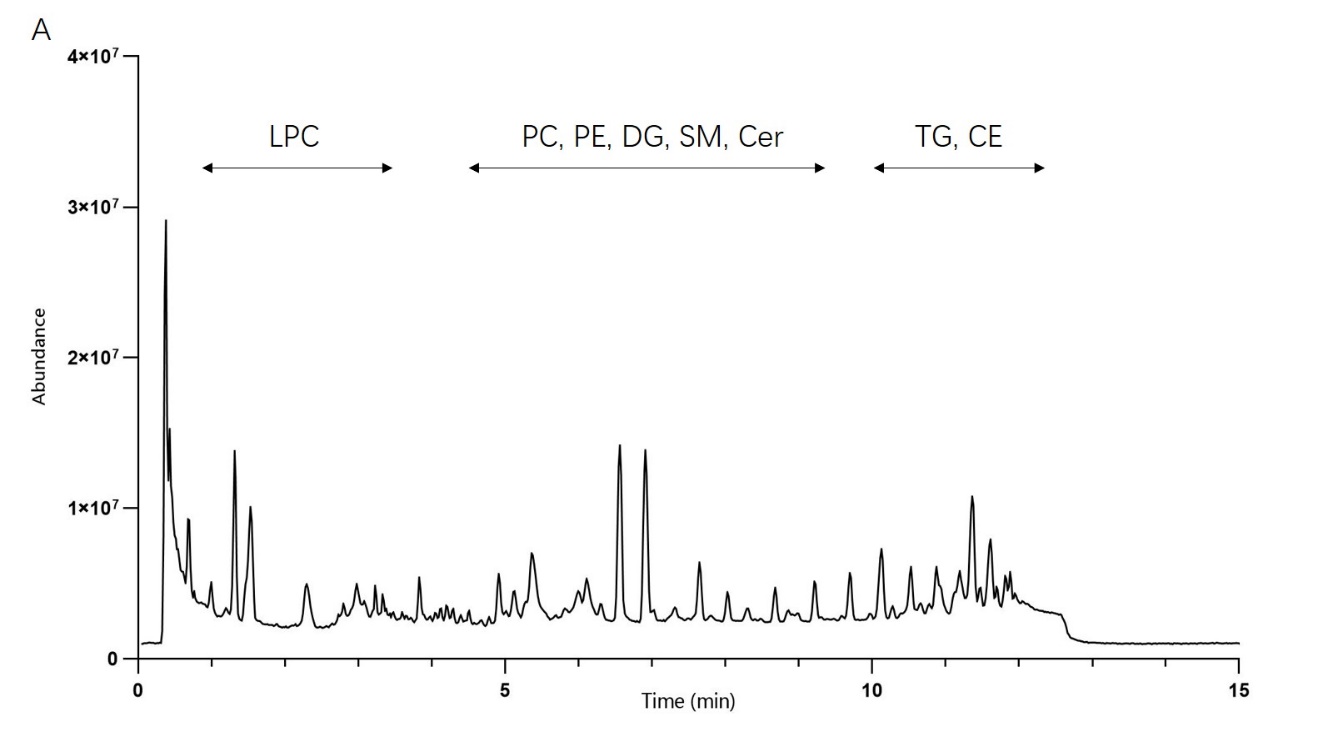


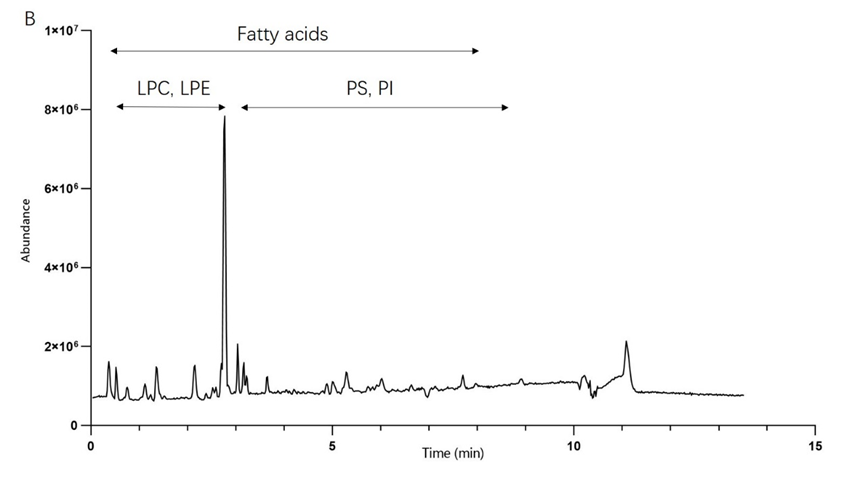


**Figure S1. (A) LC-MS positive TIC diagram of rats plasma; (B) LC-MS negative TIC diagram of rats plasma**

**Table S1. MS-based identification of the lipid molecular species detected in the present study.**

| **RT**  **(min)** | **Lipids** | **Adduct** | **m/z** | **Error**  **(ppm)** | **MSMS** | **CG vs. MG** | | | **MG vs. DG** | |
| --- | --- | --- | --- | --- | --- | --- | --- | --- | --- | --- |
|  |  |  |  |  |  | **VIP** | **P value** | **FC** | **P value** | **FC** |
| 0.96 | Testosterone | [M+NH4]+ | 306.2432 | 1.43 | 67.0554, 163.1115, 224.0731 | 1.55 | 2.6E-03 | 0.72 | 8.2E-05 | 1.83 |
| 1.18 | LPC (20:5) | [M+H]+ | 542.3236 | 1.00 | 86.0967, 104.1072, 184.0729 | 1.41 | 5.1E-03 | 1.38 | 2.0E-01 | 0.84 |
| 2.76 | LPE (22:0) | [M+H]+ | 538.3863 | 0.71 | 86.0976, 327.1956, 397.2413 | 1.70 | 8.2E-04 | 0.75 | 9.0E-03 | 1.25 |
| 4.78 | PC (34:3) | [M+H]+ | 756.5551 | 1.66 | 86.0960, 124.9969, 184.0719, 520.3485 | 1.84 | 2.2E-05 | 2.19 | 1.0E-03 | 0.56 |
| 4.78 | PC (36:5) | [M+H]+ | 780.5546 | 1.01 | 86.0974, 124.9990, 184.0726, 225.2318 | 1.85 | 2.0E-05 | 2.43 | 1.7E-04 | 0.47 |
| 4.89 | PC (38:7) | [M+H]+ | 804.5561 | 2.92 | 86.0966, 184.0721 | 1.76 | 1.4E-04 | 1.68 | 5.7E-02 | 0.76 |
| 4.91 | PC (36:4) | [M+H]+ | 782.5718 | 3.04 | 86.0963, 184.0728, 478.3307 | 1.81 | 7.1E-05 | 2.03 | 3.0E-02 | 0.70 |
| 4.93 | SM (34:1) | [M+H]+ | 703.5750 | 0.07 | 184.0721, 86.0964 | 1.57 | 5.3E-04 | 1.76 | 2.2E-02 | 0.80 |
| 4.98 | PC (33:2) | [M+H]+ | 744.5540 | 0.30 | 86.0939, 124.9979, 184.0746 | 1.81 | 6.6E-05 | 1.72 | 1.7E-02 | 0.70 |
| 5.14 | PC (32:1) | [M+H]+ | 732.5545 | 0.92 | 125.0027, 184.0720, 490.2645 | 1.75 | 1.0E-04 | 2.32 | 8.0E-05 | 0.45 |
| 5.23 | PC (36:4) | [M+H]+ | 782.5694 | 0.03 | 86.0966, 104.1071, 124.9994,  184.0728, 502.3251 | 1.88 | 1.2E-05 | 1.93 | 7.6E-03 | 0.72 |
| 5.23 | PC (38:7) | [M+H]+ | 804.5525 | 1.66 | 86.0965, 184.0725 | 1.83 | 1.9E-05 | 2.40 | 5.3E-02 | 0.75 |
| 5.33 | PC (34:2) | [M+H]+ | 758.5720 | 3.47 | 86.0955, 184.0725, 124.9973, 643.5136 | 2.00 | 5.1E-08 | 2.45 | 2.0E-03 | 0.68 |
| 5.33 | SHexCer (t34:1) | [M+H]+ | 796.5275 | 4.49 | 86.0968, 613.4629, 737.4456 | 1.97 | 3.5E-07 | 2.26 | 1.4E-03 | 0.65 |
| 5.58 | PC (37:4) | [M+H]+ | 796.5831 | 2.55 | 86.0972, 124.9967, 184.0716 | 1.59 | 1.9E-03 | 1.43 | 2.2E-04 | 0.59 |
| 5.66 | SM (36:1) | [M+H]+ | 731.6033 | 3.96 | 83.0536, 184.0723, 239.1472, 557.4796 | 1.64 | 3.6E-04 | 2.14 | 1.0E-01 | 0.81 |
| 5.71 | PC (O-36:4)/  PC (P-36:3) | [M+H]+ | 768.5899 | 0.36 | 86.0993, 184.0731 | 1.91 | 1.5E-06 | 1.88 | 4.2E-03 | 0.75 |
| 5.76 | PC (32:0) | [M+H]+ | 734.5704 | 1.34 | 184.0728, 313.2782, 479.3395, 647.5101 | 1.93 | 8.0E-07 | 1.82 | 3.3E-03 | 0.79 |
| 5.76 | PC (O-38:5)/  PC (P-38:4) | [M+H]+ | 794.6050 | 1.04 | 184.0758, 506.3563, 310.1409, 663.5281 | 1.96 | 2.0E-07 | 1.76 | 1.8E-01 | 0.89 |
| 5.86 | PC (34:1) | [M+H]+ | 760.5856 | 0.67 | 184.0717, 672.5170, 417.2981, 313.2728 | 1.71 | 1.4E-04 | 1.65 | 7.9E-03 | 0.75 |
| 5.94 | PC (38:4) | [M+H]+ | 810.6013 | 0.72 | 86.0965, 124.9990, 184.0724 | 1.73 | 1.7E-04 | 1.73 | 4.3E-02 | 0.77 |
| 5.94 | PC (40:7) | [M+H]+ | 832.5837 | 1.67 | 86.0958, 104.1059, 124.9996, 184.0718 | 1.72 | 1.9E-04 | 2.01 | 3.2E-02 | 0.71 |
| 6.06 | PC (36:2) | [M+H]+ | 786.6026 | 2.43 | 86.0968, 125.0004, 184.0735, 603.5226 | 1.83 | 2.2E-05 | 1.84 | 3.6E-03 | 0.68 |
| 6.44 | PC (O-38:4)/  PC (P-38:3) | [M+H]+ | 796.6216 | 0.07 | 86.0974, 184.0727 | 1.87 | 3.2E-06 | 1.60 | 3.7E-02 | 0.85 |
| 6.53 | PC (34:0) | [M+H]+ | 762.6000 | 0.95 | 86.0957, 104.1106, 184.0719 | 1.52 | 1.6E-03 | 1.26 | 4.4E-01 | 0.95 |
| 6.61 | PC (36:1) | [M+H]+ | 788.6240 | 9.66 | 125.0008, 184.0713, 417.2985 | 1.65 | 2.9E-04 | 1.47 | 1.7E-02 | 0.82 |
| 6.78 | PC (38:2) | [M+H]+ | 814.6335 | 1.83 | 86.0962, 124.9988, 184.0721 | 1.88 | 2.5E-06 | 1.97 | 1.3E-01 | 1.19 |
| 7.21 | SM (42:2) | [M+H]+ | 813.6847 | 0.35 | 86.0970, 124.9982, 184.0729 | 1.56 | 9.9E-04 | 1.52 | 6.1E-02 | 0.85 |
| 7.58 | DG (34:3) | [M+H]+ | 591.4964 | 3.17 | 97.1025, 195.1227, 313.2723, 335.2574 | 1.41 | 2.0E-02 | 0.49 | 6.2E-05 | 2.01 |
| 7.63 | SM (d41:1) | [M+H]+ | 801.6829 | 1.92 | 184.0736, 603.5290, 759.6426 | 1.54 | 3.6E-03 | 1.26 | 7.5E-01 | 1.02 |
| 7.64 | Cer (36:3) | [M+H]+ | 562.5188 | 1.01 | 282.2822, 459.3250 | 1.78 | 1.6E-04 | 0.87 | 6.4E-01 | 1.03 |
| 7.64 | Cer (38:3) | [M+H]+ | 590.5497 | 1.74 | 59.0590, 89.0586, 107.0456 | 1.66 | 1.1E-03 | 0.89 | 1.0E-01 | 1.11 |
| 8.29 | DG (34:0) | [M+NH4]+ | 614.5694 | 3.95 | 97.1030, 313.2724, 341.3039 | 1.36 | 2.7E-02 | 0.50 | 3.0E-04 | 1.89 |
| 8.31 | DG (36:3) | [M+H]+ | 619.5259 | 6.03 | - | 1.34 | 3.2E-02 | 0.35 | 4.9E-04 | 3.09 |
| 10.76 | TG (50:4) | [M+NH4]+ | 844.7385 | 0.53 | 599.5013, 573.4803, 827.6992 | 1.27 | 3.0E-02 | 2.25 | 2.0E-02 | 0.40 |
| 10.89 | TG (54:6) | [M+NH4]+ | 896.7697 | 0.56 | 575.5029, 577.5124, 599.4959 | 1.32 | 2.1E-02 | 2.44 | 2.8E-02 | 0.42 |
| 10.94 | CE (20:5) | [M+NH4]+ | 688.6038 | 1.59 | 217.1896, 287.2788, 369.3512, 489.3781 | 1.42 | 6.5E-03 | 1.40 | 7.0E-03 | 0.64 |
| 11.23 | TG (53:4) | [M+NH4]+ | 886.7778 | 9.04 | 869.8354, 589.5257, 601.5120 | 1.22 | 4.8E-02 | 1.54 | 2.3E-02 | 0.58 |
| 11.28 | TG (54:5) | [M+NH4]+ | 898.7845 | 1.47 | 881.7523, 601.5190, 337.2737 | 1.23 | 4.3E-02 | 1.98 | 3.5E-02 | 0.47 |
| 11.28 | TG (56:8) | [M+H]+ | 903.7369 | 7.43 | 337.2626, 547.4034, 7475.6158 | 1.52 | 3.6E-03 | 1.86 | 1.2E-02 | 0.58 |
| 11.58 | TG (54:6) | [M+H]+ | 879.7424 | 1.42 | 95.0853, 577.5220, 597.4831 | 1.24 | 4.9E-02 | 1.37 | 2.3E-02 | 0.69 |
| 11.63 | TG (54:4) | [M+NH4]+ | 900.8020 | 0.50 | 545.4810, 599.4999, 603.5330, 883.7724 | 1.24 | 3.9E-02 | 1.95 | 3.4E-02 | 0.48 |
| 4.40 | PI (36:4) | [M-H]- | 857.5224 | 4.47 | 241.0111, 255.2322, 297.0369,  303.2332, 553.2769 | 2.00 | 1.0E-04 | 1.56 | 4.2E-01 | 0.92 |
| 4.45 | PI (34:2) | [M-H]- | 833.5225 | 4.73 | 223.0005, 241.0111, 255.2322,  279.0384, 553.2729 | 2.34 | 3.9E-06 | 2.28 | 4.5E-02 | 0.77 |
| 4.48 | PI (38:5) | [M-H]- | 883.5367 | 2.80 | 223.0018, 241.0127, 255.2324,  303.2342, 579.2923 | 1.67 | 3.0E-03 | 1.37 | 2.0E-01 | 0.85 |
| 4.92 | PS (39:3) | [M-H]- | 826.5642 | 4.58 | 168.0419, 279.2323, 504.3081, 766.5372 | 2.29 | 1.5E-04 | 1.94 | 4.7E-02 | 0.70 |
| 5.00 | PI (38:4) | [M-H]- | 885.5529 | 3.36 | 222.9994, 241.0097, 303.2302, 581.3127 | 1.30 | 2.6E-02 | 1.28 | 8.4E-01 | 0.98 |
| 5.08 | PI (36:2) | [M-H]- | 861.5537 | 4.44 | 223.0004, 241.0110, 279.2323,  297.0363, 315.0487, 581.3095 | 2.28 | 2.6E-05 | 2.23 | 2.0E-02 | 0.72 |
| 5.23 | PS (39:3) | [M-H]- | 826.5644 | 4.79 | 168.0421, 255.2323, 279.2325,  303.2323, 766.5375 | 2.41 | 2.6E-06 | 1.71 | 6.6E-01 | 0.96 |
| 5.93 | PS (41:3) | [M-H]- | 854.5952 | 4.12 | 168.0419, 283.2637, 303.2323,  508.3441, 794.5684 | 2.07 | 1.8E-04 | 1.43 | 1.0E-01 | 0.87 |
| 6.03 | PS (39:1) | [M-H]- | 830.5952 | 4.18 | 168.0419, 279.2323, 508.3393, 770.5685 | 2.34 | 9.1E-06 | 1.42 | 9.7E-03 | 0.83 |
| 6.42 | PS (40:1) | [M-H]- | 844.6115 | 4.96 | 168.0409, 279.2308, 297.2793, 784.5792 | 1.99 | 5.2E-04 | 1.33 | 1.1E-01 | 0.87 |

FC: Fold Change, means raw abundance of group (2)/group (1); > 1 and < 1 means increased or decreased, respectively.

LPC: Lysophosphatidylcholine; LPE: Lysophosphatidylethanolamine; PC: Phosphatidylcholine; PC (O/P): Plasmalogen; SHexCer: Sulfatide; SM: Sphingomyelin; Cer: Ceramide; DG: Diacylglycerol; TG: Triacylglycerol; PI: Phosphatidylinositol; PS: Phosphatidylserine.

**Table S2. Chemical similarity enrichment analysis of differential metabolites**

| Cluster name | Cluster size | *P* values | FDR | Altered metabolites | Increased | Decreased | Increased ratio |
| --- | --- | --- | --- | --- | --- | --- | --- |
| Phosphatidylcholine | 18 | 3.7E-17 | 2.2E-16 | 18 | 18 | 0 | 1 |
| Triglyceride | 7 | 6.8E-10 | 2.0E-09 | 7 | 7 | 0 | 1 |
| Phosphatidylserine | 5 | 4.6E-05 | 9.3E-05 | 5 | 5 | 0 | 1 |
| Phosphatidylinositol | 5 | 7.6E-05 | 1.1E-04 | 5 | 5 | 0 | 1 |
| Sphingomyelin | 4 | 3.6E-04 | 4.3E-04 | 4 | 4 | 0 | 1 |
| Plasmalogen | 3 | 5.1E-04 | 5.1E-04 | 3 | 3 | 0 | 1 |

*P* values are the result of the Kolmogorov Smirnov test evaluating how significant difference a metabolite cluster was represented by chance. FDR is the Benjamini Hochberg corrected *p* values. The Increase or Decreased shows the numbers of increased or decreased significant compounds in a cluster. The Increased ratio means Increased compouds/total Altered metabolites.

**Table S3. Disturbed metabolic pathways in BSS**

| Pathway | Total | Hits | Raw *p* | Holm *p* | FDR | Impact |
| --- | --- | --- | --- | --- | --- | --- |
| Glycerophospholipid metabolism | 36 | 6 | 1.8E-07 | 1.5E-05 | 1.5E-05 | 0.20 |
| Sphingolipid metabolism | 21 | 3 | 6.1E-04 | 5.0E-02 | 2.6E-02 | 0.27 |
| Glycerolipid metabolism | 16 | 2 | 7.7E-03 | 6.3E-01 | 2.2E-01 | 0.10 |
| Phosphatidylinositol signaling system | 28 | 2 | 2.3E-02 | 1.0E+00 | 4.4E-01 | 0.12 |
| Inositol phosphate metabolism | 30 | 2 | 2.6E-02 | 1.0E+00 | 4.4E-01 | 0.08 |
| Linoleic acid metabolism | 5 | 1 | 4.2E-02 | 1.0E+00 | 5.9E-01 | 0.00 |
| alpha-Linolenic acid metabolism | 13 | 1 | 1.1E-01 | 1.0E+00 | 1.0E+00 | 0.00 |
| Glycosylphosphatidylinositol anchor biosynthesis | 14 | 1 | 1.1E-01 | 1.0E+00 | 1.0E+00 | 0.00 |
| Ether lipid metabolism | 20 | 1 | 1.6E-01 | 1.0E+00 | 1.0E+00 | 0.18 |
| Arachidonic acid metabolism | 36 | 1 | 2.7E-01 | 1.0E+00 | 1.0E+00 | 0.00 |
| Steroid biosynthesis | 42 | 1 | 3.1E-01 | 1.0E+00 | 1.0E+00 | 0.00 |
| Steroid hormone biosynthesis | 77 | 1 | 5.0E-01 | 1.0E+00 | 1.0E+00 | 0.05 |

The Total is the total number of compounds in the pathway; the Hits is the actually matched number from the user uploaded data; the Raw *p* is the original *p* value calculated from the enrichment analysis; the Holm *p* is the *p* value adjusted by Holm-Bonferroni method; the FDR is the *p* value adjusted using False Discovery Rate; the Impact is the pathway impact value calculated from pathway topology analysis.

**Table S4. The typical components and related targets in *Salvia Miltiorrhiza* (Danshen)**

| Components | Formula | Pubchem ID | Targets counts | Targets |
| --- | --- | --- | --- | --- |
| Caffeic acid | C9H8O4 | 689043 | 4 | Akr1b1, Alox5, Alox15, Alox15b |
| Ferulic acid | C10H10O4 | 445858 | 4 | Akr1b1, Alox5, Ptgs2, Hsd17b3 |
| Isoferulic acid | C10H10O4 | 736186 | 4 | Akr1b1, Alox5, Ptgs2, Hsd17b3 |
| Lithospermic acid | C27H22O12 | 6441498 | 2 | Akr1b1, Ptgs2 |
| Luteolin | C15H10O6 | 5280445 | 11 | Cyp19a1, Soat1, Soat2, Akr1b1, Ptgs2, Alox5, Akr1a1, Pla2g1b, Pla2g2a, Alox15, Alox15b |
| Protocatechualdehyde | C7H6O3 | 8768 | 1 | Comt |
| Protocatechuic acid | C7H6O4 | 72 | 2 | Comt, Alox5 |
| Rosmarinic acid | C18H16O8 | 5281792 | 4 | Akr1b1, Alox15, Alox15b, Alox5 |
| Salvianolic acid A | C26H22O10 | 5281793 | 1 | Akr1b1 |
| Salvianolic acid B | C36H30O16 | 6451084 | 1 | Akr1b1 |
| Salvianolic acid D | C20H18O10 | 75412558 | 1 | Akr1b1 |
| Salvianolic acid E | C36H30O16 | 86278266 | 1 | Akr1b1 |
| Salvianolic acid F | C17H14O6 | 10903113 | 3 | Akr1b1, Alox5, Ptgs2 |
| Salvianolic acid H | C27H22O12 | 21582559 | 1 | Akr1b1 |
| Salvianolic acid I | C27H22O12 | 10459878 | 1 | Akr1b1 |
| Succinic acid | C4H6O4 | 1110 | 1 | Akr1d1 |
| Sugiol | C20H28O2 | 94162 | 3 | Alox5, Cyp19a1, Dgka |
| Tanshinaldehyde | C19H18O4 | 124268 | 6 | Pik3ca, Pik3r1, Akr1b1, Ptgs2, Alox5, Akr1a1 |
| Tormentic acid | C30H48O5 | 73193 | 6 | Hsd3b1, Lpl, Pla2g1b, Cyp17a1, Fdft1, Ptgs2 |
| Ursolic acid | C30H48O3 | 64945 | 6 | Lss, Pla2g1b, Inppl1, Enpp6, Ptgs2, Cyp17a1 |

**Table S5. Functional enrichment result of potential targets**

| Pathway | Potential targets | Counts | *P* value | FDR |
| --- | --- | --- | --- | --- |
| Steroid hormone biosynthesis | Comt, Cyp17a1, Cyp19a1,  Hsd3b1, Hsd17b3, Akr1d1 | 6 | 3.20E-06 | 3.60E-03 |
| Arachidonic acid metabolism | Alox15, Alox15b, Alox5,  Pla2g1b, Pla2g2a, Ptgs2 | 6 | 3.20E-06 | 3.60E-03 |
| Ovarian steroidogenesis | Alos5, Cyp17a1, Cyp19a1, Hsd3b1, Ptgs2 | 5 | 2.00E-05 | 2.20E-02 |
| Steroid biosynthesis | Fdft1, Lss, Soat1, Soat2 | 4 | 2.50E-05 | 2.80E-02 |
| Glycerolipid metabolism | Akr1a1, Akr1b1, Dgka, Lpl | 4 | 6.70E-04 | 7.40E-01 |
| Phosphatidylinositol signaling system | Dgka, Inppl1, Pik3ca, Pik3r1 | 4 | 2.70E-03 | 3.00E+00 |
| Serotonergic synapse | Alox5, Alox15, Alox15b, Ptgs2 | 4 | 5.60E-03 | 6.10E+00 |
| Linoleic acid metabolism | Alox15, Pla2g1b, Pla2g2a | 3 | 6.40E-03 | 6.90E+00 |
| Ether lipid metabolism | Enpp6, Pla2g1b, Pla2g2a | 3 | 7.40E-03 | 7.90E+00 |
| Regulation of lipolysis in adipocytes | Pik3ca, Pik3r1, Ptgs2 | 3 | 1.30E-02 | 1.40E+01 |
| VEGF signaling pathway | Pik3ca, Pik3r1, Ptgs2 | 3 | 1.30E-02 | 1.40E+01 |
| B cell receptor signaling pathway | Inppl1, Pik3ca, Pik3r1 | 3 | 1.80E-02 | 1.80E+01 |
| Prolactin signaling pathway | Cyp17a1, Pik3ca, Pik3r1 | 3 | 2.10E-02 | 2.10E+01 |
| Fc gamma R-mediated phagocytosis | Inppl1, Pik3ca, Pik3r1 | 3 | 2.60E-02 | 2.50E+01 |
| Small cell lung cancer | Pik3ca, Pik3r1, Ptgs2 | 3 | 2.70E-02 | 2.60E+01 |
| Ras signaling pathway | Pik3ca, Pik3r1, Pla2g1b, Pla2g2a | 4 | 3.00E-02 | 2.90E+01 |
| Glycerophospholipid metabolism | Dgka, Pla2g2a, Pla2g2a | 3 | 3.20E-02 | 3.00E+01 |
| Choline metabolism in cancer | Dgka, Pik3ca, Pik3r1 | 3 | 3.60E-02 | 3.30E+01 |

Counts represent the total numbers of potential targets involved in the corresponding pathways; the *P* value is the original *p* value calculated from the enrichment analysis; the FDR is the *p* value adjusted using False Discovery Rate.
